# Combining commercial assessment products with administrative data to shape education policy: Tracking pupil outcomes during the Covid-19 pandemic

**DOI:** 10.23889/ijpds.v8i2.2322

**Published:** 2023-09-14

**Authors:** Jon Andrews

**Affiliations:** 1 Education Policy Institute, London, United Kingdom

## Objectives

To inform the government’s response to the COVID-19 pandemic by providing an ongoing analysis of the learning loss and recovery experienced by pupils in England after the cancellation of national statutory assessments. Our aim was to use data from a commercial assessment product linked to large administrative datasets.

## Methods

We constructed a pupil level dataset of assessments in reading and mathematics. The assessments covered multiple time points in the pandemic period and in the years before. We matched this to the National Pupil Database to give a wide range of pupil characteristics information – including eligibility for free school meals, the area they lived in, and information about their school.

By constructing a regression model of pupil outcomes in the pre-pandemic period, we produced a counterfactual of what we would have expected each pupil to achieve had the pandemic not occurred and hence estimate the extent of learning loss.

## Results

National lockdowns and restrictions to in-person learning were associated with pupils in England making less progress than normal. By the summer of 2021, learning losses in reading amongst primary-aged pupils amounted to around 0.9 months of learning, and learning losses amongst secondary-aged pupils amounted to around 1.8 months. Learning losses in mathematics were even greater, at 2.8 months. We found that the pandemic’s effects were not felt evenly with larger losses for those from economically disadvantaged backgrounds and larger losses in parts of the north and midlands than in London and the south. Indeed, by the start of the 2021/22 academic year, results in reading for primary aged pupils in London and the south-west were broadly in line with pre-pandemic levels.

## Conclusion

The project demonstrated the power of combining commercial products with administrative datasets to shape policy. The analysis informed the government’s education response to the pandemic including the targeting of additional support to pupils from low-income backgrounds who, as subsequent national tests have confirmed, were disproportionately affected by the pandemic.

